# Human embryonic and neuronal stem cell markers in retinoblastoma

**Published:** 2007-06-08

**Authors:** Gail M. Seigel, Abigail S. Hackam, Arupa Ganguly, Lorrie M. Mandell, Federico Gonzalez-Fernandez

**Affiliations:** 1 Ross Eye Institute, University of Buffalo, Buffalo, NY; 2 Department of Ophthalmology, University of Buffalo, Buffalo, NY; 3 University of Miami, Bascom Palmer Eye Institute, Miami, FL; 4 Department of Genetics, University of Pennsylvania School of Medicine, Philadelphia, PA; 5 Department of Pathology, University of Buffalo, Buffalo, NY; 6 Medical Research Service, Veterans Affairs, Buffalo, NY

## Abstract

**Purpose:**

Retinoblastoma (RB) is the most common intraocular tumor of early childhood. The early onset of RB, coupled with our previous findings of cancer stem cell characteristics in RB, led us to hypothesize that subpopulations of RB tumors harbor markers and behaviors characteristic of embryonic and neuronal origin.

**Methods:**

Our RB sources included: human pathological tissues, and the human RB cell lines Y79 and WERI-RB27. Microarray screening, single and dual-label immunocytochemistry and RT-PCR were performed to detect embryonic and neuronal stem cell markers, such as Oct3/4, Nanog, CD133, and Musashi-1. To test for functional evidence of stem cell behavior, we examined RB cells for their ability to form neurospheres and retain BrdU label as indicators of self-renewal and slow cell cycling, respectively.

**Results:**

Microarray comparisons of human RB tumors with normal retinal tissue detected upregulation of a number of genes involved in embryonic development that were also present in Y79 cells, including Oct3/4, Nanog, Musashi-1 and Musashi-2, prominin-1 (CD133), Jagged-2, Reelin, Thy-1, nestin, Meis-1,NCAM, Patched, and Notch4. Expression of Musashi-1, Oct3/4 and Nanog was confirmed by immunostaining and RT-PCR analyses of RB tumors and RB cell lines. CD133 expression was confirmed by PCR analysis. Y79 and WERI-RB27 contained populations of Hoechst-dim/ABCG2-positive cells that co-localized with embryonic stem cell markers Oct3/4-ABCG2 and Nanog-ABCG2. Subpopulations of Y79 and WERI-RB27 cells were label-retaining (as seen by BrdU incorporation) and were able to generate neurospheres, both hallmarks of a stem cell phenotype.

**Conclusions:**

Small subpopulation(s) of RB cells express human embryonic and neuronal stem cell markers. There are also subpopulations that demonstrate functional behavior (label retention and self-renewal) consistent with cancer stem cells. These findings support the hypothesis that RB is a heterogeneous tumor comprised of subpopulation(s) with stem cell-like properties.

## Introduction

Retinoblastoma is the most common intraocular childhood tumor with an estimated annual incidence of approximately four per one million children. Retinoblastoma occurs in both germline (40%) and sporadic (60%) forms [1]. When retinoblastoma is confined to the eye, more than 90% of patients will be cured of the primary tumor. One present challenge in the treatment of retinoblastoma is metastatic and secondary tumors that reduce life span and quality of life [2,3]. One potential hypothesis for the appearance of subsequent tumors in RB is the persistence of cancer stem cells [4,5], small subpopulations of cells believed to be primarily responsible for tumor progression as well as resistance to chemotherapy [6] and radiation treatments [7]. The presence of such cells, which have been identified in a number of cancers, have yet to be proven in retinoblastoma.

Stem-like cells are reported to exist in a number of malignancies, including leukemias [8,9], brain tumors [10,11], breast cancer [12-14] lung cancer [15], prostate cancer [16], as well as in cancer cell lines [17]. As shown in our previous study [18], retinoblastoma tumors and cell lines also contain small subpopulations of cells that display stem cell characteristics. In RB, these stem cell characteristics include the presence of a side population [19], (ie. cells that are able to exclude Hoechst 33342 dye based on expression of the drug transporter/stem cell marker ABCG2), and expression of other stem cell markers such as ALDH1, MCM2, and SCA-1 [18]. In the present study, we sought to expand upon our previous findings and define the nature of the RB stem cells using a combination of gene expression and functional analyses. We chose to examine unsorted, heterogeneous populations of RB cells to identify potential subpopulations for further study. To this end, we tested the hypothesis that RB tumors contain subpopulation(s) of cells that retain characteristics consistent with stem cells of human embryonic and neuronal origin.

## Methods

### Human tissues

Human tissues were obtained according to the guidelines of the Declaration of Helsinki and our IRB-approved protocol. Four cases of human retinoblastoma of varying classification, that had been removed by enucleation, were prepared and examined as either paraffin or frozen sections. Since most archival cases of human RB are preserved in paraffin, we were limited in the number of human cases that we could examine due to the inability of Oct3/4 and Nanog antibodies to recognize their targets in paraffin-embedded tissues, despite attempts at antigen retrieval. Therefore, we made no correlations between tumor staging/histological characteristics and immunoreactivity of human embryonic stem cell markers. Four additional microdissected human RB tumors were used for RT-PCR analysis. Three additional human RB tumors were used for microarray analysis and compared with normal retinal tissue.

### Cell lines

Long-term human retinoblastoma cell lines were also used for this study, specifically Y79 cells [20] and WERI-RB27 cells [21]. WERI-Rb27 cells were grown in DMEM with 10% calf serum, 1X MEM nonessential amino acids (GIBCO, Grand Island, NY), 1X MEM vitamins (GIBCO), 0.37% sodium bicarbonate, 0.058% l-glutamine and 100 μg/ml gentamicin. Y79 cells were grown in RPMI 1640 with 10% fetal calf serum, 0.37% sodium bicarbonate, 0.058% l-glutamine, 10 mM HEPES, and 100 μg/ml gentamicin.

### BrdU incorporation

The WERI-27 and Y79 cells were grown in standard medium, as described above and pulsed with 10 μM BrdU for 7 days and washed out for 14 days. The cells were the fixed with 4% paraformaldehyde, washed with PBS, and incubated with 500 μl 1 mg/ml DNAse I (Sigma, St. Louis, MO) in PBS for 30 min at 37 °C. After a second wash in PBS the cells were resuspended in 100 μl PBS/0.1% BSA/ 0.5% Nonidet-P40. The BrdU antibody (BD Pharmingen) was added at 5 μg/ml and the samples were incubated for 45 min at room temperature. The samples were washed twice and resuspended with PBS/0.1% BSA and TRITC-labeled anti-mouse IgG (Sigma) was added at 27 μg/ml. Samples were incubated in the dark for 30 min at room temperature and then washed twice with PBS/0.1% BSA. Cells were loaded onto a slide with a coverslip and analyzed using fluorescence imaging. The brightest cells were counted.

### Neurosphere assay

WERI-RB27 and Y79 cells were plated in a 96 well dish at densities from 1,000 cells per well to 50 cells per well. To promote neurosphere formation rather than reaggregation, the cells were plated at very low density (50 cells per well) so that there would be a low probability of the cells encountering one another in the well. After 5 days, wells were examined for neurosphere formation and the neurospheres were counted. For continuous passage, initial plating density was 100 cells per well. Neurospheres were counted and then dissociated into single cell suspensions, diluted 1:2, and replated.

### Immunohistochemistry

Primary antibodies, their origins and working concentrations are presented in Table 1. Frozen sections of human tumors were processed as follows: Cryosections were rinsed in PBS. Goat serum (5%) was used for blocking of nonspecific staining on slides. Slides were incubated in 0.25% Triton X-100 for 5 min. After a rinse in PBS, sections were incubated for 1 h with primary antibody as per concentrations listed in Table 1. After rinsing three times for 5 min each in PBS, slides were incubated with 1 μg/ml of biotinylated goat anti-rabbit or anti-mouse immunoglobulin (Zymed/Invitrogen, Carlsbad, CA) for 60 min. Slides were incubated for 20 min with horseradish peroxidase-conjugated avidin (Elite kit, Vector Laboratories, Burlingame, CA). The slides were rinsed in 0.05 M Tris (pH 7.4) and the final immunoreaction proceeded with diaminobenzidine (Vector). The dark brown reaction product was viewed by light microscopy. Negative control tissues were incubated in 5% isotype control serum instead of primary antibody and did not generate reaction product.

**Table 1:** Primary antibodies used for immunostaining.

**Antibody target**	**Specificity**	**Company (catalog number**	**Concentration**
ABCG2	human, mouse	Signet Labs (BXP-21)	6.25 μg/ml
Alkaline phosphatase	human, mouse	R&D Systems MAB1448	10 μg/ml
Oct3/4	human	R&D Systems AF1759	5 μg/ml
Nanog	human	R&D Systems AF1997	10 μg/ml
Musashi-1	human	Neuromics RA14128	15 μg/ml
BrdU	human	BD Pharmingen 555627	5 μg/ml

The following is a list of commercially-available primary antibodies used for immunocytochemistry.

### Immunocytochemistry

Suspensions of Y79 and WERI-RB27 cells were examined for co-localization of ABCG2, embryonic stem cell markers Oct3/4 or Nanog, as well as Hoechst dye exclusion. Live cells were incubated with 5 μg/ml Hoechst 33342 dye for 15 min, rinsed in PBS, and gently pelleted for antibody staining. Primary antibodies ABCG2 and Oct 3/4 or Nanog were co-incubated at the working concentrations (listed in Table 1) for one hour. Cells were pelleted at low speed, rinsed in PBS, and incubated in fluorescent secondary antibody (TRITC-secondary antibody for ABCG2; FITC-secondary antibody for Oct3/4 or Nanog) for one hour. Cells

were post-fixed in 4% paraformaldehyde, washed twice with PBS, resuspended, then pipetted onto a slide and coverslipped for microscopic viewing. Fluorescent cells were visualized with a Nikon ES600 microscope with epifluorescent filters for Hoechst and TRITC. Digital images were captured with a SONY ICX 285AL SPOT camera (Diagnostic Instruments, Sterling Heights, MI).

### Reverse transcriptase polymerase chain reaction

One microgram of total RNA from the four human RB tissues and the cell lines was reversed transcribed into cDNA using Thermoscript (Invitrogen) followed by incubation with RNase H for 30 min at 37 °C, according to the manufacturer's procedures. The stem cell genes were amplified from undiluted cDNA samples using PCR with 45 s denaturation at 94 °C, 45 s annealing at 58-62 °C and 60 s extension at 72 °C. A negative control that did not have reverse transcriptase was used to identify amplification from contaminating genomic DNA, and a no template control was included for each reaction. The PCR primers were designed to span an intron. Primer sequences are listed in Table 2.

**Table 2:** Primers used for reverse transcriptase polymerase chain reaction.

**Gene**	**Primer (5'-3')**
*OCT3/4*	F: AGTGAGAGGCAACCTGGAGA
	R: CAAAAACCCTGGCACAACT
*Nanog*	F: CAAAGGCAAACAACCCACTT
	R: ATTGTTCCAGGTCTGGTTGC
*Msi1*	F: GAGACTGACGCGCCCCAGCC
	R: CGCCTGGTCCATGAAAGTGACG
*Prom-1 CD133)*	F: TGGATGCAGAACTTGACAACGT
	R: ATACCTGCTACGACAGTCGTGGT

The following primers were used for reverse transcriptase polymerase chain reaction. F represents forward; R represents reversed.

### Microarray analysis

The analysis of human RB tumors vs. normal retinal tissue (A.G.) was conducted independently from the Y79 microarray analysis (A.S.H.). Results from the two separate studies were correlated and compared for presentation in Table 3.

**Table 3:** Human embryonic genes upregulated in human retinoblastoma tumors and expressed in Y79 cells.

**Gene**	**Gene ID**	**Avg. fold-change Log2**	**Avg. FDR value**
*Oct 3/4*	NM_002701.3	1.19	0.61
*Nanog*	NM_024865	1.24	0.518
*Musashi-1*	NM_002442	1.14	0.765
*Musashi-2*	NM_138962.2	1.028	0.513
*MCM2*	NM_004526.2	3.56	0.777
*Meis-1*	NM_002398.2	1.37	0.606
*Nestin*	NM_006617.1	0.68	0.172
*Reelin*	NM_005045.2	1.12	0.766
*Jagged-2*	NM_145159.1	1.68	0.764
*NCAM-1*	NM_181351.1	1.37	0.636
*Prominin-1 (CD133)*	NM_006017.1	1.4	0.583
*Thy-1*	NM_006288.2	1.2	0.479
*Patched*	NM_000264	1.14	0.659
*Notch-4*	NM_004557.3	1.12	0.454

Microarray analysis was used to compare human retinoblastoma (RB) tumors (n=3) with normal retinal tissue (n=3) Human embryonic genes of interest that were upregulated as compared with normal retina were cross-referenced with independently-derived microarray data from Y79 retinoblastoma cells. The genes listed in this table represent human embryonic stem cell genes that are upregulated in RB (as compared with normal retina) and are also present in Y79 cells.

### Human retinoblastoma tumors

Samples of retina and the adjoining retinoblastoma were obtained from eyes enucleated at the Will's Eye Hospital (WEH) as part of a research collaboration with Dr. Carol Shields. This work was done according to IRB approved protocols at WEH and University of Pennsylvania. Total RNA was isolated from three pairs of fresh frozen samples and hybridized to HG133 version 2.0 chips from Affymetrix, CA. Differential expression of genes expressed in stem cell lineages was followed by PaGE analysis [22]. The False Discovery Rate (FDR) is the expected percent of false predictions in a set of microarray data. For example if the algorithm returns 100 genes with a false discovery rate of 0.3 then we should expect 70 of them to be correct and 30 of them to be incorrect. More information on false discovery rates can be found at UPENN.

### Y79 cells

Total RNA was isolated from proliferating Y79 cells using Trizol phenol-based extraction (Invitrogen), as described in [23]. The quality of the RNA was assessed by gel electrophoresis and A_260_/A_280_ ratio. Microarray analyses were performed by Ocean Ridge Biosciences (Jupiter, FL) using six human HEEBO (human exonic-evidence based-oligonucleotide) 70-mer oligonucleotide microarrays, containing approximately 50,000 probes (representing exonic sequences, alternatively spliced exonic, ESTs and controls). Biotinylated UTP complementary RNA (cRNA) probes were prepared, fragmented and hybridized to the microarrays for 16-18 h with constant rotation. The microarray slides were washed under stringent conditions, stained with Streptavidin-Alexa-647 (Invitrogen), and scanned using an Axon GenePix 4000B scanner.

For data analyses, the local background was subtracted and the spot intensities were log_2_-transformed. The values were then normalized by subtraction of the array mean and the spots were filtered by threshold. Based on the signal from the negative control probes, a threshold was calculated for intensity level cut-offs for which 99% of the negative controls fall below those cut-offs.

## Results

In the present study, we examined unsorted, heterogeneous populations of RB cells in order to identify subpopulation(s) with human embryonic and neuronal stem cell properties.

### Human embryonic genes upregulated in human retinoblastoma tumors are also detected in the Y79 cell line

To identify stem cell genes that are differentially expressed in RB, three human RB tumors of varying grade were compared with surrounding normal retinal tissue by Affymetrix microarray analysis. We identified stem cell genes that were upregulated in human RB tumors as compared with surrounding retinal tissue with a FDR (false discovery rate) value of at least 0.1. In turn, stem cell genes that were upregulated in human RB tumors were cross-referenced against independently-obtained microarray data of stem cell genes detected as "present above threshold intensity levels" in Y79 retinoblastoma cells. The stem cell genes that overlapped, based on these criteria, are shown in Table 3. The fold changes are presented as Log2. The greatest fold-change between RB and normal retina was for MCM2, a neural stem cell marker that we first demonstrated as being immunoreactive in RB tumors [18], and was reported to be more highly expressed in more highly invasive RB tumors [24]. Many of the fold changes in stem cell genes were rather small, most likely due to the small contribution of the stem-like cell population (about 1%) to the sum total of gene expression.

Microarray analysis of Y79 human retinoblastoma cells was performed to confirm the expression stem cell marker genes in retinoblastoma using a different microarray platform. RNA was obtained from 6 independent plates of sub-confluent cells. Comparisons of the Y79 microarray results revealed that many of the stem cell markers upregulated in human RB were also found in the RB cell line (extra upregulated in human RB tumors). These include: Oct3/4, Nanog, Musashi-1 and Musashi-2, prominin-1 (CD133), Jagged-2, Reelin, Thy-1, nestin, Meis-1, NCAM, Patched, and Notch4. We selected the genes from Table 3 that were the most closely associated with embryonic development, namely Oct3/4, Nanog and Musashi-1 for further confirmation and analysis.

### Detection of Oct 3/4, Nanog, Musashi-1, and CD133 in human retinoblastoma tumors, retinoblastoma cell lines, and normal retina by reverse transcriptase polymerase chain reaction

**Figure 1:**
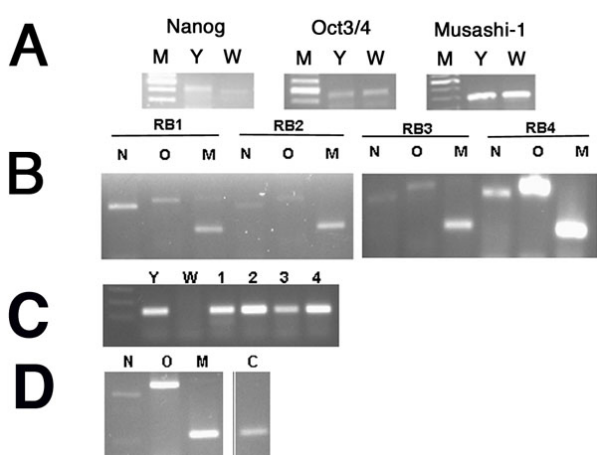
Detection of Oct 3/4, Nanog Musashi-1, and CD133 in human retinoblastoma tumors, retinoblastoma cell lines and normal human retina by reverse transcriptase polymerase chain reaction. Human retinoblastoma (RB) tumors and cell lines were examined by RT-PCR as described in Methods. **A**: Nanog, Oct3/4, and Musashi gene expression in WERI-RB27 and Y79 cell lines. M represents marker; Y represents Y79; W represents WERI-RB27. **B**: Nanog (N), Oct3/4 (O), and Musashi-1 (M) expression in four human RB tumors (RB1, 2, 3, 4). **C**: CD133 expression seen in Y79 cells (Y) and RB tumors (1,2,3,4), but not WERI-RB27 cells (W). **D**: Nanog (N), Oct3/4 (O), Musashi-1 (M) and CD133 (C) expression in normal human retina.

Human embryonic stem cell markers Oct3/4 and Nanog are genes of pluripotency and self-renewal. In Figure 1, Oct3/4, Nanog, and Musashi-1 were detected by PCR analysis of human retinoblastoma cells, tumors, and normal retina. Since the original cell populations and tumor samples represented mixed populations of stem-like cells and non-stem-like cells, we did not attempt to quantitate differences in expression levels between RB tumors and cell lines. Instead, we sought to determine the presence or absence of gene expression. As seen in Figure 1A, Nanog, Oct3/4 and Musashi-1 gene expression was evident in both Y79 cells and WERI-RB27 cells. In Figure 1B, the same genes are present in four human RB tumors. In Figure 1C, CD133 expression was present in Y79 cells and RB tumors, but not WERI-RB27 cells. In Figure 1D, Nanog, Oct3/4 and Musashi expression is shown in normal retinal tissues at low levels. These results confirm the microarray findings.

### Oct 3/4 and Nanog immunoreactivity in human retinoblastoma tumors

**Figure 2:**
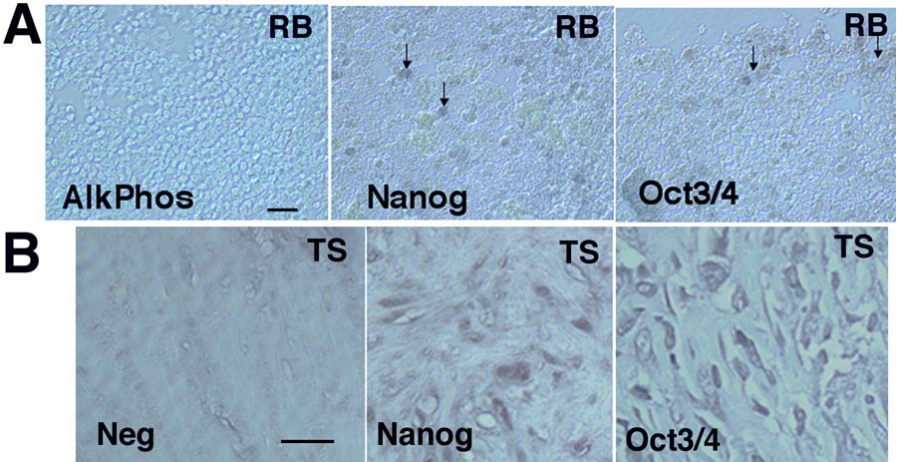
Human retinoblastoma tumors exhibit immunoreactivity to human embryonic stem cell markers Oct 3/4 and Nanog. **A**: Human retinoblastoma (RB) tumors were prepared as frozen sections, and immunostained for human embryonic stem cell markers AlkPhos, Oct3/4, and Nanog, Immunoreactivity to Oct3/4 and Nanog was evident (eg. arrows), while no reaction was seen AlkPhos. **B**: Human testicular seminoma tissue (TS) was used as a positive control for Oct3/4 and Nanog. The scale bars represents 5 μm.

As further evidence of gene expression at the protein level, human RB tumors were examined for immunoreactivity to Oct3/4 and Nanog. Non-necrotic areas of the tumors were examined to avoid potential areas of high background staining. As seen in Figure 2, small populations of RB cells within the RB tumors were immunoreactive for Oct3/4 and Nanog. As positive controls, we tested human testicular seminomas for Oct3/4 and Nanog expression. These results are shown in Figure 2B.

### Musashi-1 expression in retinoblastoma tumors and cell lines

**Figure 3:**
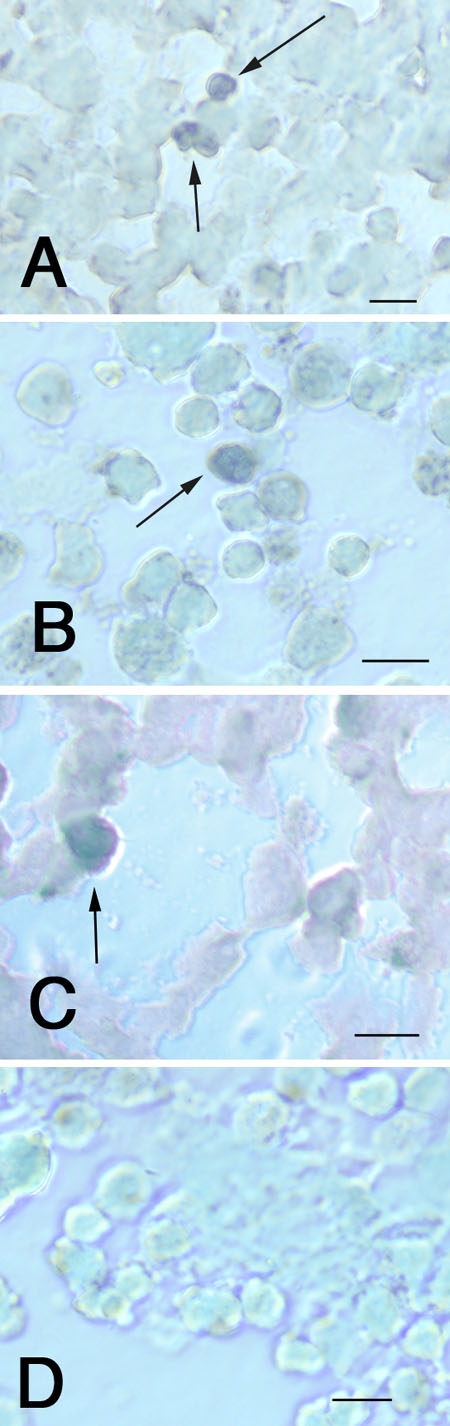
Musashi-1 in retinoblastoma tumors and cell lines. Human retinoblastoma (RB) tumors and cell lines were prepared as frozen sections, and immunostained for Musashi-1: **A**: Human RB tumor; **B**: Y79 cells; **C**: WERI-RB27 cells; **D**: Negative control. The scale bar represents 5 μm. Arrows indicate positive cells.

Musashi-1 is hypothesized to play a role in cell fate determination, differentiation and tumorigenesis through its influence on the Notch signaling pathway. The pattern of Musashi-1 immunoreactivity in human RB tumors (frozen and paraffin-embedded), as well as RB cell lines Y79 and WERI-RB-27 is shown in Figure 3. In all cases, small numbers of immunoreactive cells were present in both RB tumors and cell lines.

### Colocalization of Nanog or Oct 3/4 with Hoechst-dim/ABCG2 positive cells

**Figure 4:**
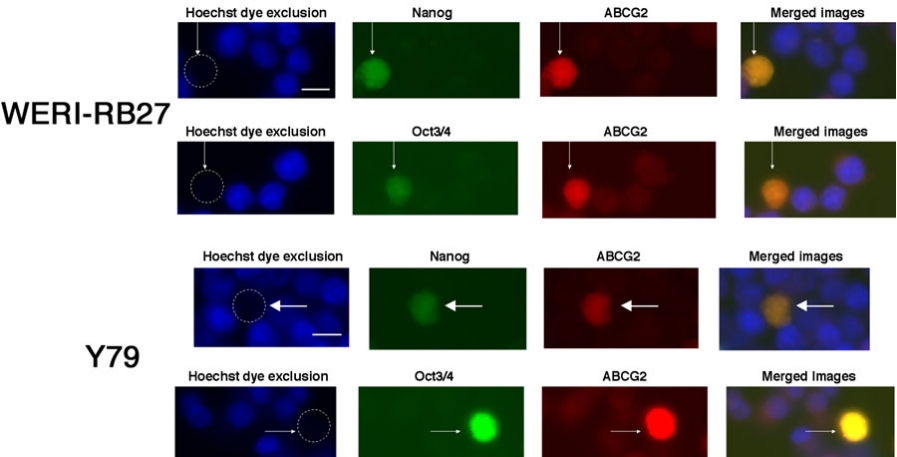
Colocalization of Nanog or Oct 3/4 in Hoechst-dim/ABCG2 positive cells. Y79 and WERI-RB27 human retinoblastoma cells were examined for fluorescent Hoechst 33342 dye uptake, ABCG2 immunoreactivity, coupled with either Nanog or Oct3/4 immunoreactivity. Each horizontal panel depicts the same microscopic field, viewed under separate fluorescent filters for Hoechst, FITC and TRITC, as well as a merged image of all three fields. As seen in the "Hoechst dye exclusion" field, the arrow points to a cell that has excluded the Hoechst dye and appears "Hoechst dim". This is due to the active Hoechst dye exclusion properties of the ABCG2 protein. In the next two panels, we see the same cell, as indicated by the arrow, that is immunoreactive for Nanog or Oct3/4 and ABCG2. When the three images are merged, ABCG2 colocalizes with both Nanog and Oct 3/4. The scale bar represents 5 μm.

Since we had previously shown a small subpopulation of Hoechst-dim/ABCG2 positive cells in RB [18], we sought to examine whether these same cells would co-express the embryonic stem cell markers Oct3/4 and Nanog. As seen in Figure 4, ABCG2-bright, Hoechst-dim cells co-localize with Oct3/4 and Nanog. This result was demonstrated in both the Y79 and WERI-RB27 cell lines.

### Subpopulations of retinoblastoma cells are slow-cycling

**Figure 5:**
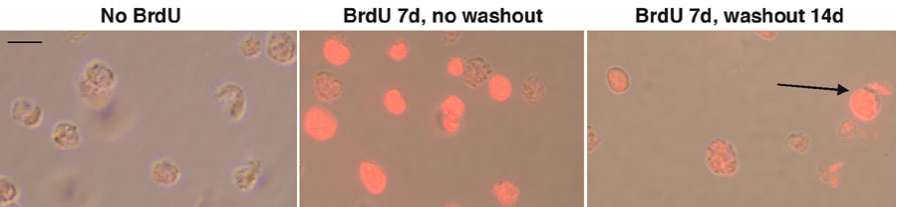
Label-retaining cells in retinoblastoma cultures. Y79 human retinoblastoma cells were pulsed with 10 μM BrdU for 4-7 days and washed out for 14 days. At 14 days, BrdU-immunoreactive cells comprised approximately 3-4% of the population. **A**: No BrdU added (negative control); **B**: BrdU added for 7 days without washout. **C**: BrdU for 7 days and 14 days of washout. Three percent of cells were still labeled after 14 days (arrow). The scale bar represents 5 μm.

One of the hallmarks of stem cell growth is slow cell cycling [25]. Cell cycling can be measured by incorporation of bromodeoxyuridine (BrdU), followed by wash-out with medium lacking BrdU. Slowly-cycling cells retain BrdU longer after washout than quickly-cycling cells. In Figure 5, Y79 human retinoblastoma cells were pulsed with 10 μM BrdU for 7 days and washed out for 14 days. The brightest cells above background were counted as positive for BrdU label retention. At 14 days, BrdU-immunoreactive cells comprised 3% of the population, an indication of slow cell cycling and further evidence of the presence of stem cell-like cells in the population. This 3% figure is greater than the percentage of cells (less than 1%) that we detected in the side population previously [18]. Therefore, there may be slow-cycling cells that fall outside of the side population.

### Neurosphere formation in retinoblastoma cultures as a sign of self-renewal

**Figure 6:**
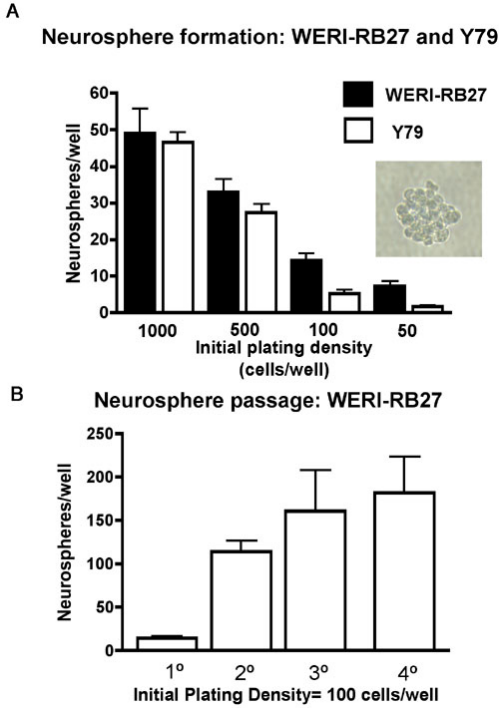
Neurosphere formation in retinoblastoma cultures. **A**: Y79 and WERI-R27 cells were plated as single cell suspensions in 96 well dishes at initial plating densities of 50-1,000 cells per well, in triplicate. Low cell densities were chosen to minimize effects of non-specific cell aggregation in favor of neurospheres originating from one single cell. After five days, neurospheres were counted and results presented. Both Y79 and WERI-RB27 human retinoblastoma cells formed neurospheres at all cell densities tested. A typical neurosphere (WERI-RB27) is shown (inset). **B**: WERI-RB27 cells were prepared as single cell suspensions at an initial plating density of 100 cells per well, in triplicate. Every 3-4 days, neurospheres were counted and then dissociated into single cell suspensions, diluted 1:2, and replated. The graph depicts the number of neurospheres counted at each passage.

We examined Y79 and WERI-RB27 human retinoblastoma cells for their capacity to form neurospheres, an indicator of stem cell self-renewal [26]. Cells were plated as single cell suspensions in 96 well dishes at initial plating densities of 50-1,000 cells per well, in triplicate. Low cell densities were chosen to minimize effects of non-specific cell aggregation in favor of neurospheres originating from one single cell. After five days, neurospheres were counted and results presented, as shown in Figure 6. Both Y79 and WERI-RB27 human retinoblastoma cells were capable of neurosphere formation at all cell densities tested. Both the Y79 and WERI-RB27 cell lines were able to generate neurospheres at low cell density, a hallmark of stem cell self-renewal. Neurosphere formation was higher at the lowest two densities in Weri compared with Y79 cells. Furthermore, neurospheres could be repeatedly passaged (Figure 6B).

## Discussion

Human embryonic stem cell markers are expressed in developing embryos, germ cell tumors [27] and breast carcinomas [28]. In the present study, we have shown that RB subpopulations express human embryonic stem cell genes involved in stem cell pluripotency and self-renewal, such as Oct 3/4, Nanog and Musashi-1. Furthermore, the RB cell lines show functional properties of stem cell populations. The expression of pluripotency genes such as Oct 3/4 and Nanog would suggest a broad range of differentiation options for these stem-like subpopulations of cells. However, the full differentiation potential of these stem-like cell populations remains unknown.

The presence of early embryonic genes in RB may contribute to our understanding about the RB cell of origin. Dyer and Bremner [29] have proposed either a "progenitor cell model" or a "transition cell model" in which epigenetic and/or genetic differences in individual RB cells could lead to tumor formation upon inactivation of the RB1 tumor suppressor gene. In this study, we have detected subpopulations of RB cells that are immunoreactive for human embryonic and neuronal stem cell markers. Considering the early childhood diagnosis of RB, this phenomenon raises questions regarding the persistence of stem cells of embryonic origin in RB tumors and the possibility that an embryonic stem cell may be responsible for the initiation of RB tumors. Our results suggest that either amplification of a resident stem cell population or reversion of retinal cells to a stem-like state may be associated with the development of RB.

A number of stem cell genes are expressed in RB and are of interest based upon the stem cell characteristics that they are known to confer. ABCG2 corresponds with a Hoechst-33342-negative phenotype of side population (SP) cells [19]. ABCG2 is expressed in leukemia [30], germ cell cancers [31], as well as cancers of the breast [32], prostate [16], and lung [33]. Our previous study demonstrated expression of ABCG2 along with the neural stem cell marker MCM2 in RB cells and tumors [18]. Expression of ABCG2 and MCM2 has been associated with increased tumor invasiveness in retinoblastoma [24]. The present findings illustrate that ABCG2 is not only expressed in Hoechst-dim RB cells, but is co-localized with human embryonic stem cell markers, such as Oct3/4 and Nanog as further indication of a stem-like cell phenotype.

Musashi-1 is an RNA binding protein linked to asymmetric cell division and expressed in brain tumors and breast cancer [34,35]. Oct-3/4 is a POU transcription factor that is associated with self-renewal and pluripotency of stem cells [36]. Oct3/4 has been associated with tumorigenesis of adult germ cells. Ectopic expression of Oct3/4 in mice leads to formation of dysplastic skin and intestinal lesions due to an increase in the number of progenitor cells [37]. Ours is the first report of Oct3/4 expression in a CNS tumor of non-germ cell origin.. This is also the first report of the homeodomain gene Meis1 associated with retinoblastoma. Meis1 is known to be coexpressed with homeobox genes, such as HOXA5, 7 and 9 in myelogenous leukemias [38]. Patched, implicated in the carcinogenesis of medulloblastoma [39], reelin, expressed in normal retinal development and in response to tissue injury [40], were also found to be upregulated in RB in our microarray analysis. The upregulation of Notch4 indicates that the Notch signaling pathway, important in breast development/cancer [41] may be a potential target for novel RB therapies. Our findings of stem cell markers upregulated in RB that share characteristics with other cancers may help identify common mechanisms among divergent types of cancer stem cells in terms of gene expression and stem cell phenotypes.

Nanog, another human embryonic stem cell marker, is also a transcription factor. Overexpression of Nanog in human embryonic stem cells promotes pluripotency and allows propagation over multiple passages [42], whereas knockdown of Nanog induces differentiation into mature cell types [43]. Nanog has been proposed as a diagnostic marker for germinomas of the central nervous system [44]. Greater than 90% of CNS germinomas exhibit Nanog expression, whereas tumors considered in the differential diagnosis of germinomas do not [44]. In light of our results, retinoblastoma is another CNS tumor that exhibits both Oct3/4 and Nanog expression.

CD133 (Prominin-1) is a cell surface marker of cancer stem cells [45,46]. In addition, CD133 expression was previously reported for both Y79 and WERI-RB1 cells [47]. Interestingly, a frameshift mutation of CD133 at codon 614 has been associated with human retinal degeneration, whereas native CD133 protein is localized to photoreceptors [48]. Therefore, CD133 appears to be an important component in photoreceptor maintenance and/or function. In our study, independent microarray analyses of both the Y79 cell line and RB tumors demonstrated the presence of CD133 and an upregulation of CD133 in RB tumors, as compared with surrounding retinal tissues. In glioblastoma, CD133+ cells were significantly more resistant to chemotherapy agents such as carbaplatin, taxol, and etoposide than CD133 negative cells [45]. Therefore, the extent of CD133 expression, in addition to expression of the ABCG2 drug transporter, may be another factor to consider with regard to chemotherapy resistance in retinoblastoma.

In brain tumors, nestin-positive cells are associated with a perivascular cancer stem cell niche [49]. In human medulloblastoma, ependymoma, oligodendroglioma and glioblastoma, tumors with the highest concentrations of blood vessels exhibited the greatest number of Nestin+ cells. Furthermore, these same cells were located proximal to blood vessels within the tumors [49]. From the present study, nestin expression is upregulated in RB over normal retinal tissue and expressed in Y79 cells. The proximity of nestin-positive cells to blood vessels in RB tissues remains to be determined over a large number of samples.

We cannot make conclusions as to the overlap between the various stem cell markers and behaviors in RB, aside from our data that ABCG2-positive cells co-localize Nanog and Oct3/4. Are these the same cells that initiate neurospheres or retain BrDU? Further investigations are necessary to determine whether the subpopulation of stem-like cells represent yet another heterogeneous population. The ability of particular stem cell-like subpopulations to form tumors in animals will be an important test of the cancer stem cell phenotype.

It is interesting to note that a number of human embryonic and neuronal markers are present in normal human retina. It is known that retinal progenitors exist in the ciliary margin [50], These retinal progenitor cells may contribute to our detection of stem cell markers in normal retina after nucleic acid amplification in PCR analyses. However our microarray analysis does show that these genes, although present in normal retinal tissue, are upregulated in RB tumors, as shown in Table 3.

The presence of embryonic stem cell markers in RB has clinical implications. It appears that RB tumors contain subpopulations of cells that possess sufficiently unique properties that would allow them to survive chemotherapy and retain their tumor-forming potential. The identification of a subpopulation of cancer stem cells that drives tumorigenesis and chemo-resistance in retinoblastoma may lead to new approaches for determining prognosis and optimal therapy. Expression patterns of stem cell markers, especially CD133 [45], may indicate the differentiated state of retinoblastoma tumors, and may correlate with a favorable/unfavorable prognosis in the clinical setting. Human RB tumors express a variety of multi-drug transporters with the ability to confer resistance to chemotherapy [51,52]. New agents designed to efficiently kill or terminally differentiate these RB subpopulations may lead to more effective treatments for both primary tumors and metastases.
